# Enhanced immunosurveillance for animal morbilliviruses using vesicular stomatitis virus (VSV) pseudotypes

**DOI:** 10.1016/j.vaccine.2016.10.010

**Published:** 2016-11-11

**Authors:** Nicola Logan, William G. Dundon, Adama Diallo, Michael D. Baron, M. James Nyarobi, Sarah Cleaveland, Julius Keyyu, Robert Fyumagwa, Margaret J. Hosie, Brian J. Willett

**Affiliations:** a MRC-University of Glasgow Centre for Virus Research, Garscube Estate, Glasgow G61 1QH, UK; b Animal Production and Health Laboratory, International Atomic Energy Agency, Seibersdorf, Austria; c The Pirbright Institute, Pirbright, Woking, Surrey GU24 0NF, UK; d Boyd Orr Centre for Population and Ecosystem Health, Institute of Biodiversity Animal Health and Comparative Medicine, University of Glasgow, Glasgow G12 8QQ, UK; e Tanzania Wildlife Research Institute (TAWIRI), Arusha, Tanzania

**Keywords:** Morbillivirus, Peste des petits ruminants, PPRV, rinderpest, RPV, Neutralising antibody, Surveillance

## Abstract

The measurement of virus-specific neutralising antibodies represents the “gold-standard” for diagnostic serology. For animal morbilliviruses, such as peste des petits ruminants (PPRV) or rinderpest virus (RPV), live virus-based neutralisation tests require high-level biocontainment to prevent the accidental escape of the infectious agents. In this study, we describe the adaptation of a replication-defective vesicular stomatitis virus (VSVΔG) based pseudotyping system for the measurement of neutralising antibodies against animal morbilliviruses. By expressing the haemagglutinin (H) and fusion (F) proteins of PPRV on VSVΔG pseudotypes bearing a luciferase marker gene, neutralising antibody titres could be measured rapidly and with high sensitivity. Serological responses against the four distinct lineages of PPRV could be measured simultaneously and cross-neutralising responses against other morbilliviruses compared. Using this approach, we observed that titres of neutralising antibodies induced by vaccination with live attenuated PPRV were lower than those induced by wild type virus infection and the level of cross-lineage neutralisation varied between vaccinates. By comparing neutralising responses from animals infected with either PPRV or RPV, we found that responses were highest against the homologous virus, indicating that retrospective analyses of serum samples could be used to confirm the nature of the original pathogen to which an animal had been exposed. Accordingly, when screening sera from domestic livestock and wild ruminants in Tanzania, we detected evidence of cross-species infection with PPRV, canine distemper virus (CDV) and a RPV-related bovine morbillivirus, suggesting that exposure to animal morbilliviruses may be more widespread than indicated previously using existing diagnostic techniques.

## 1 Introduction

Once the scourge of European, African and Asian livestock and wild ruminants, rinderpest virus (RPV) is only the second virus in history after smallpox to be eradicated by vaccination. RPV is a morbillivirus, a close relative of measles virus (MeV) and peste des petits ruminants (PPRV). Like rinderpest, PPR is now being considered for global eradication by vaccination [1]. PPR has spread over the last decade and is now endemic in many areas of Africa, the Middle East, Central and Southern Asia and into China. PPRV causes a devastating disease in small ruminants, threatening both food security and the livelihoods of smallholders [2], [3]. As such, PPR has been selected as a top priority disease to be addressed by the World Organization for Animal Health (OIE), with a global plan for eradication by 2030 [4].

Goats vaccinated with RPV-derived vaccines resist PPRV infection [5], [6], [7]. For example, following vaccination with a vaccinia RPV recombinant, the vaccinates initially produced an RPV-specific response prior to challenge, with no cross-reactive PPRV antibodies [7]. However, following challenge with PPRV and elicitation of a PPRV-specific response, the RPV-specific response increased significantly, consistent with the PPRV challenge boosting the RPV-specific response [7]. Similarly, PPRV vaccines elicit both PPRV and RPV neutralising antibodies [8] and measles vaccines cross-prime an anti-canine distemper virus (CDV) response in dogs [9]. While the induction of cross-neutralising antibodies is a common feature of exposure to morbilliviruses, the strength and specificity of the response varies between viral species.

The “gold standard” for measuring morbillivirus-specific antibodies is the virus neutralisation test [10]. However, classical tests for neutralising antibodies are restricted to viral strains that are amenable to propagation in cell culture. During the adaptation of the virus for growth in cell culture, the biological properties of the virus may alter dramatically to suit the new *ex vivo* environment and the availability of potential viral receptors [11], [12], [13], [14], [15], [16], [17], [18], [19]. As the receptor binding domains of the morbilliviral haemagglutinins are targets for neutralising antibodies [20], alterations in receptor binding that facilitate infection *in vitro* may alter the conformation of the viral haemagglutinin, modulating sensitivity to neutralising antibodies. If biologically-relevant neutralising antibody responses are to be quantified accurately, assays that utilize primary field strains of virus and their cognate receptors are required. By generating viral “pseudotypes” bearing both the haemagglutinin (H) and fusion (F) proteins of the morbillivirus [11], [21], neutralising antibody responses may be measured against primary, field isolates of the morbillivirus. Moreover, as viral pseudotype-based neutralisation assays are not constrained by the ability of the primary morbillivirus to grow in the target cell, inter-assay variability is minimized. Finally, where the viruses being studied present a significant biohazard, pseudotype-based assays circumvent the need for high-level bio-containment.

In this study we examined the neutralisation of ruminant morbilliviruses by sera from animals exposed to either PPRV or RPV. We demonstrate that while cross-neutralisation is evident between the two morbilliviruses, the strength and breadth of the response against the two viruses differs markedly. Further, we identify an RPV-specific neutralising activity, indicative of the circulation of an RPV-related bovine morbillivirus.

## 2 Materials and methods

### 2.1 Cell lines and sera

HEK293 [22] and HEK293T cells were maintained in Dulbecco’s Modified Eagle Medium (DMEM) supplemented with 10% foetal bovine serum, 100 IU/ml penicillin, 100 μg/ml streptomycin, 2 mM glutamine and 0.11 mg/ml sodium pyruvate. Media for 293T cells and 293 cells stably expressing canine SLAM were supplemented with 400 μg/ml G418. All media and supplements were obtained from Life Technologies Ltd., Paisley, UK. Sera were collected from ruminants exposed to PPRV, RPV or PPRV and RPV vaccines. Samples from cattle vaccinated with live attenuated PPRV, RPV, and wild type PPRV have been described previously [23]. Miscellaneous sera from goats, sheep and cattle infected with either PPRV or RPV were obtained from The Pirbright Institute, Surrey, UK and the Joint United Nations Food and Agriculture Organization (FAO) and International Atomic Energy Agency (IAEA) Biotechnology and Agriculture Laboratories (Joint FAO/IAEA), Seibersdorf, Austria.

### 2.2 Eukaryotic expression vectors and recombinant viruses

The recombinant vesicular stomatitis virus (VSV) in which the glycoprotein (G) gene has been deleted (VSVΔG) and replaced with firefly luciferase (*luc*) has been described [24], [25] and was kindly provided by Michael Whitt, Memphis, TN, USA. The construction of the RPV H and F expression plasmids and those for the vaccine strain of PPRV (PPRV/Nigeria/75/1) has been previously described [23]. Primary PPRV H and F cDNAs were amplified from viral RNA. PPRV containing supernatants from cultures of PPRV isolates Senegal 1969 [26] (lineage I), Benin 2010 [27] (lineage II), Kenya 2011 [28] (lineage III) and Ethiopia 2010 (lineage IV, Joint FAO/IAEA PPRV Bank, Seibersdorf, Austria) were mixed with RLT lysis buffer (QIAgen) and RNA prepared as per manufacturer’s instructions using a QIAamp Viral RNA Mini kit (QIAgen). This RNA was then used to prepare first strand cDNA (Transcriptor First Strand cDNA Synthesis Kit, Roche) and used as a template in PCR reactions using Q5 high fidelity DNA polymerase (see Supplementary Methods for details of primers). Amplification products were digested with *Sal*I and *Not*I and cloned into the vector VR1012 (Vical Inc.). The nucleic acid sequences of all amplified cDNAs were determined externally (*LIGHTrun* Sequencing Service, GATC Biotech AG, Cologne, Germany). All oligonucleotide primers were obtained from Integrated DNA Technologies, Leuven, Belgium.

### 2.3 VSVΔG*luc* pseudotype preparation

293T cells were transfected with the H and F expression vectors from the respective morbillivirus, followed by super-infection with VSVΔG*luc* (VSVG) as described [24], [25]. Supernatants were harvested and titred on 293dogSLAM cells [24], [25]. Luciferase activity was measured by the addition of Steadylite plus™ (Perkin Elmer) and a Microbeta 1450 Jet luminometer (Perkin Elmer). The viral titre [50% tissue culture infectious dose (TCID_50_)] was calculated using the Spearman-Kärber formula [29].

### 2.4 Pseudotype-based neutralisation assay

2 × 10^4^ 293-dogSLAM cells were plated into each well of a 96-well plate (Culturplate-96, Perkin Elmer, Coventry, UK). Fourfold serum dilutions were prepared in triplicate in complete medium ranging from 1:8 to 1:32768. The diluted serum samples were then added to the 293-dogSLAM cells followed by 2.5 × 10^3^ TCID_50_ of each VSVΔG*luc* pseudotype. Luciferase activity was measured at 48–72 h post-plating and antibody titres calculated by interpolating the point at which there was a 90% reduction in luciferase activity (90% neutralisation, inhibitory concentration 90 or IC_90_) [30].

### 2.5 Live virus-based PPRV neutralisation assay

Virus neutralising activity in sera from cattle vaccinated with live attenuated RPV and PPRV vaccines was assessed in a 96-well plate based micro-neutralisation assay as per the standard OIE specified procedure [10]. The target virus was PPRV Nigeria/75/1 and the target cells were Vero dogSLAM. The neutralising titre was expressed as the reciprocal of the antibody dilution at which 50% of the wells showed virus growth.

### 2.6 PPRV cELISA

Antibodies against PPRV H in African sera were detected by ELISA using a competitive ELISA (The Pirbright Institute, Surrey and BDSL, Irvine, UK) as per the manufacturers’ instructions. Briefly, inactivated PPRV is coated onto an ELISA plate prior to the addition of test and control sera in the presence of a monoclonal antibody against PPRV H. Antibodies within the test sample compete for binding with the anti-H monoclonal antibody, binding of which is then quantified using an anti-mouse IgG horseradish peroxidase (HRP) conjugated antibody. The percent inhibition (PI) of monoclonal antibody binding for each test sample is calculated in comparison with positive and negative control sera. The diagnostic threshold for the assay is set at 50% PI of the monoclonal antibody control. A PI value of <25% is considered negative.

## 3 Results

### 3.1 Neutralisation of PPRV pseudotypes by sera from vaccinated and infected animals

**Fig. 1:**
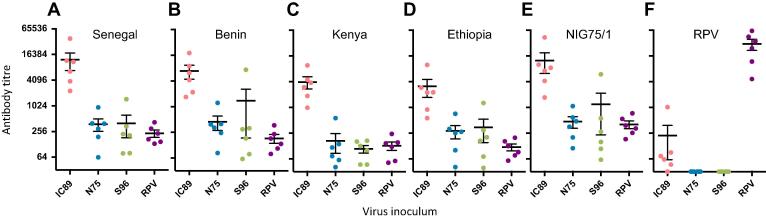
Neutralising antibody titres in cattle following PPRV or RPV inoculation. Four groups of six cattle were inoculated with PPRV/Ivory Coast/89 (IC89), PPRV/Nigeria/75/1 (N75), PPRV/Sungri/96 (S96) or RPV/RBOK (RPV). Sera were tested at 4 weeks post-infection for neutralising activity against VSV(PPRV) pseudotypes bearing the (A) Senegal 1969, (B) Benin 2010, (C) Kenya 2011, (D) Ethiopia 2010 or (E) Nigeria 75/1 strains of PPRV, or (F) Kabete O strain of RPV. Serial dilutions of each serum sample were prepared in triplicate and screened for neutralising activity against each VSV(PPRV) pseudotype. Antibody titres were calculated based on a 90% reduction of infectivity relative to the no serum control. Mean ± SEM (*n* = 6) is shown for each group.

Neutralising antibody titres were measured in sera from cattle infected previously [23] with either PPRV/Ivory Coast/89 (IC89, wild type lineage 1), PPRV/Nigeria/75/1 (N75, vaccine lineage 2), PPRV/Sungri/96 (S96, vaccine lineage 4) or the Plowright vaccine strain of RPV (RBOK). Sera were screened for ability to neutralize pseudotypes bearing the glycoproteins of the widely used PPRV vaccine strain N75, four field strains representative of lineages 1–4 (Senegal 1969, Benin 2010, Kenya 2011 and Ethiopia 2010), and RPV Kabete O. All pre-challenge sera were negative for morbillivirus neutralising antibodies (titres < 16). Sera from IC89 infected animals displayed high antibody titres against each of the five PPRV strains (Fig. 1A–E) and cross-neutralized RPV albeit less efficiently than PPRV (Fig. 1F). Sera raised against IC89 neutralized Senegal 1969 (Fig. 1A) and N75 (Fig. 1E) (lineages 1 and 2 respectively) most efficiently. Strong activity was also noted against Benin 2010 (Fig. 1B) (lineage 2). Weaker responses were detected against Kenya 2011 (Fig. 1C) and Ethiopia 2010 (Fig. 1D) (lineages 3 and 4 respectively), the titres being significantly lower than those against lineages 1 and 2 (Wilcoxon matched pairs signed rank test, p < 0.05).

Infection with the vaccine strains N75 and S96 induced substantially lower antibody titres *per se*. Titres induced by N75 infection were highest against the lineage 2 N75 (homologous strain) and Benin 2010 strains. Neutralisation of lineage 3 Kenya 2011 by sera from the N75 vaccinates (Fig. 1C) was significantly less efficient compared to homologous virus. The relatively poor neutralisation of Kenya 2011 extended to the animals infected with the lineage 4 vaccine strain S96 (Fig. 1C), titres being significantly lower than those against all other viruses (p < 0.05). While neither the N75 or S96 vaccinates developed RPV cross-neutralising antibodies, the RPV vaccinates developed PPRV cross-neutralising antibodies at levels comparable to those induced by the N75 or S96 vaccines, consistent with the cross-protection against PPRV induced by RPV vaccines [5], [6], [7].

### 3.2 Detection of neutralising antibodies in sera from ruminants in Africa

The accurate identification of PPRV-exposed animals in the field is critical to eradication campaigns and serological tests can facilitate the identification of animal reservoirs of infection in cases where viral genomic material is undetectable in blood or body fluids. We screened a panel of Tanzanian ruminant sera (goats (*C. aegagrus hircus*), cattle (*B. taurus*), buffalo (*S. caffer*) and gazelle (*G. Thomsonii* and *G. Grant*)) from areas reported previously to harbour PPRV. The buffalo and gazelle sera had been screened previously by cELISA and classed as “negative” (<50% inhibition on cELISA), the sera which tested positive by ELISA having been sent to The Pirbright Institute for further study. Buffalo sera were collected across the Serengeti National Park (SNP) from 2005 to 2012. The goat sera, collected during 2012, came from the NCA, a region that had undergone a PPRV vaccination campaign in 2011–2012.

**Fig. 2:**
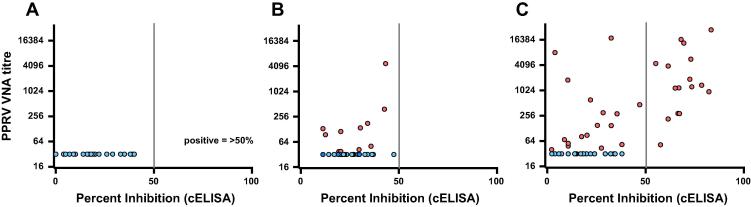
PPRV neutralising antibodies in sera from Tanzanian ruminants. Sera from (A) gazelle (*n* = 27), (B) buffalo (*n* = 42) or (C) goats (*n* = 59) were screened by competitive ELISA (cH-ELISA) for anti-PPRV H antibodies and a “percent inhibition” calculated for each sample. The samples were then re-screened for neutralising antibodies against VSV(PPRV) pseudotypes. Serial dilutions of each serum sample were prepared in triplicate and screened for neutralising activity against VSV(PPRV) pseudotypes (Nigeria/75/1). Antibody titres were calculated based on a 90% reduction of infectivity relative to the no serum control. Samples which tested negative for neutralising antibodies are shown in blue, those which tested positive for PPRV neutralising antibodies are highlighted in red. The 50% cut-off above which a sample is declared positive by the cH-ELISA is denoted by a grey line. (For interpretation of the references to color in this figure legend, the reader is referred to the web version of this article.)

The 27 gazelle sera tested negative for neutralising antibodies against PPRV, in agreement with the cELISA test findings (Fig. 2A). In contrast, 16 of the 42 buffalo sera displayed neutralising activity against PPRV (Fig. 2B), all of which had tested negative previously by cELISA. 59 goat sera were screened for neutralising antibodies, yielding 36 positive sera, 14 of which were negative by cELISA (Fig. 2C). In combination, screening of these gazelle, buffalo and goat sera suggested that the cELISA had a positive predictive value of 100%; all positives detected by cELISA harbouring neutralising antibodies. In contrast, the negative predictive value of the cELISA was 71%; 29% of ELISA negatives possessing neutralising antibodies. As the cELISA detects the competitive inhibition of anti-PPRV H monoclonal antibody binding, it is possible that the low negative predictive value reflects differences in the epitope specificity of the antiviral immune response across host species.

**Fig. 3:**
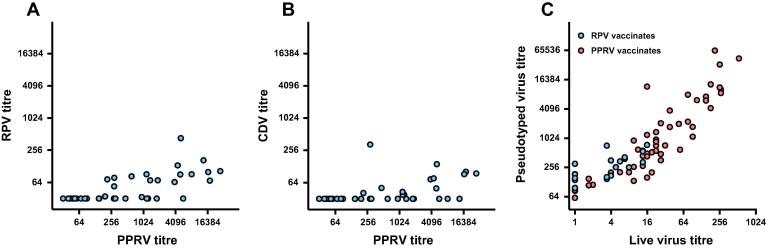
Comparison of neutralising antibody titres against VSV(PPRV) with those against VSV(RPV), VSV(CDV) or PPRV live virus. Goat sera were screened simultaneously for neutralising activity against VSV pseudotypes bearing PPRV (Nigeria/75/1) and either (A) RPV (Kabete O) or (B) CDV (Onderstepoort) glycoproteins. (C) In a separate experiment, sera from cattle vaccinated with live attenuated RPV (blue circles) or PPRV (red circles) vaccines and with known antibody titres against live PPRV Nigeria/75/1virus, were re-screened for neutralising activity against VSV(PPRV Nigeria/75/1). Antibody titres in the pseudotyped virus assay (A, B, C) were calculated based on a 90% reduction of infectivity relative to the no serum control (three replicates per dilution). Antibody titres in the live virus based assay (C) were expressed as the reciprocal of the antibody dilution at which 50% of the wells showed virus growth (six replicates per dilution). (For interpretation of the references to color in this figure legend, the reader is referred to the web version of this article.)

Next, we examined the species-specificity of the PPRV neutralising antibodies in the buffalo and goat sera, comparing PPRV titres with those against RPV and CDV. The buffalo sera did not neutralize RPV; however, 5 sera neutralized CDV, only 2 of which had anti-PPRV activity, indicative of a cross-reactive response. The remaining 3 animals recognized CDV but neither PPRV nor RPV, suggesting a primary exposure to CDV or an antigenically-related ruminant morbillivirus. In comparison with the buffalo sera, several goat sera displayed cross-neutralising activity against RPV (Fig. 3A). In general, this activity increased with anti-PPRV titre (Spearman r = 0.7366, p < 0.0001). Similar cross-reactivity was observed between the PPRV responses of the goat sera and the corresponding anti-CDV responses (Fig. 3B), albeit with a weaker correlation (Spearman r = 0.6002, p < 0.0001). Antibody titres obtained with the pseudotyped virus neutralisation assay correlated well with the titres obtained by live virus neutralisation assay (Fig. 3C) albeit with an increased sensitivity. PPRV neutralising antibody titres in 72 serum samples from cattle vaccinated with live attenuated PPRV or RPV vaccines (as described in [31] and Fig. 1) were estimated using either VSV(PPRV Nigeria/75/1) on 293-dogSLAM cells or replication competent PPRV Nigeria/75/1 virus on Vero dogSLAM cells. Titres correlated well between both assay systems (Spearman r = 0.89, p < 0.0001). The pseudotyped virus assay yielded titres approximately 100-fold higher than the “live virus”-based assay with 10 of the 72 sera that were negative by live virus neutralisation assay testing positive by the pseudotyped virus test.

### 3.3 Cross-reactivity between PPRV and RPV neutralising antibodies

**Fig. 4:**
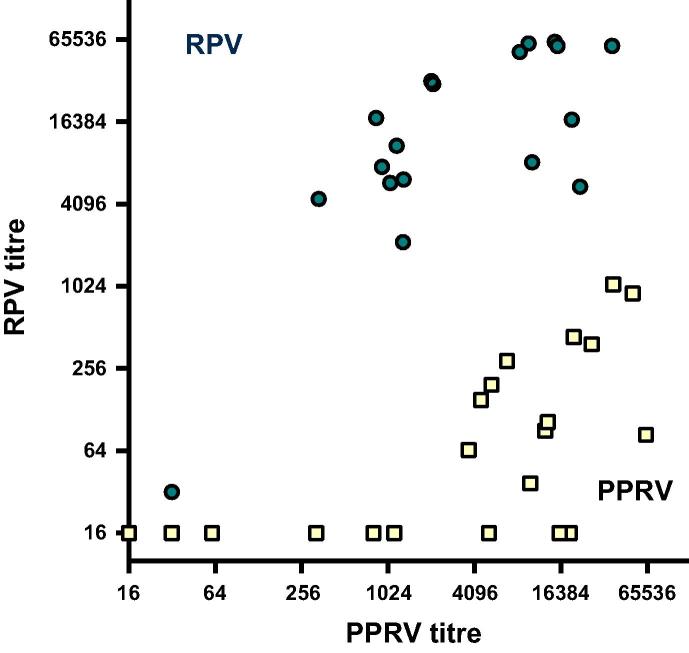
Cross-neutralisation of RPV and PPRV. Sera from animals infected with either RPV (blue circles) or PPRV (yellow squares) were screened for neutralising activity against VSV pseudotypes bearing RPV (Kabete O) or PPRV (Nigeria/75/1) glycoproteins. Serial dilutions of each serum sample were prepared in triplicate and screened for neutralising activity. Antibody titres were calculated based on a 90% reduction of infectivity relative to the no serum control. Neutralising antibody titres were highest against the homologous virus. (For interpretation of the references to color in this figure legend, the reader is referred to the web version of this article.)

Previously, we noted cross-neutralisation between the anti-PPRV and anti-CDV responses in sera from both naturally infected and vaccinated animals [32]. To characterize the degree of cross-neutralisation between PPRV and RPV, we compared sera from animals exposed to either PPRV or RPV for the presence of cross-neutralising antibodies against the heterologous virus (Fig. 4). The sera raised against RPV and PPRV clustered into two distinct groups, irrespective of the two sets of sera being derived from different host species and having been raised against a range of viral strains. Hence the RPV sera neutralized RPV more efficiently than PPRV while the PPRV sera neutralized PPRV more efficiently than RPV. Moreover, by comparing the neutralising antibody titres against PPRV and RPV, it was possible to predict whether an animal had been exposed to PPRV or RPV.

### 3.4 Detecting atypical morbillivirus infections

**Fig. 5:**
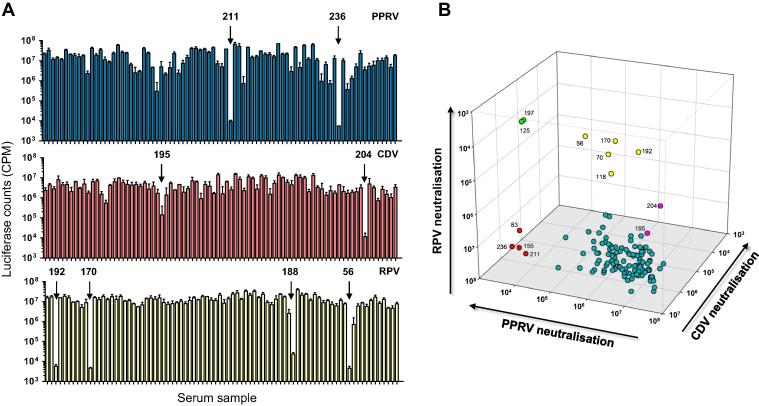
Rapid screening for atypical morbillivirus infections in Tanzanian cattle. Serum samples from 125 cattle collected in the Bunda region of Tanzania were screened for the presence of neutralising antibodies against CDV, PPRV or RPV at a single fixed dilution (1:100). VSV pseudotypes bearing the PPRV (Nigeria/75/1), CDV (Onderstepoort) or RPV (Kabete O) glycoproteins were mixed with each diluted serum sample and plated onto SLAM-expressing target cells. (A) Histograms displaying a 72 subset of samples illustrating clear discrimination of sera with >90% neutralising activity. PPRV (blue), CDV (red) and RPV (yellow). Each point represents luciferase counts per minute (CPM) at 48 h post-infection, mean ± SEM (*n* = 3). Positive samples are numbered. (B) 3D-plot comparing luciferase activity for PPRV, CDV and RPV for all 125 samples. Neutralising activity was RPV-specific (yellow circles), PPRV-specific (red circles), CDV-specific (pink) or RPV/PPRV cross-neutralising (green). (For interpretation of the references to color in this figure legend, the reader is referred to the web version of this article.)

**Fig. 6:**
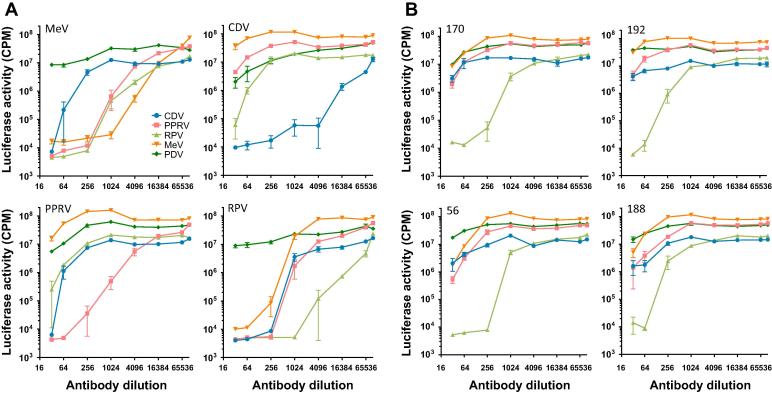
Cross-neutralising activity in sera from Tanzanian cattle. (A) VSV pseudotypes were generated bearing glycoproteins from CDV, PPRV, RPV, MeV or PDV and their sensitivity to neutralisation by sera raised against MeV, CDV, PPRV and RPV compared. (B) Sera from four Tanzanian cattle with anti-RPV activity were screened for cross-neutralisation of CDV, PPRV, MeV and PDV. RPV neutralisation was the dominant activity in the serum samples. Each neutralisation assay was repeated on three independent occasions, typical result shown. Each point represents the mean ± SEM (*n* = 3).

While PPRV is primarily a pathogen of sheep and goats, it can infect sub-clinically a range of species (reviewed in [33]). Subsequent to a PPRV outbreak in the Serengeti ecosystem in 2008, the presence of PPRV was confirmed in cattle in Northern Tanzania [34]. Previously, RPV vaccination of cattle would have induced cross-protective immunity against PPRV; however, following the global eradication of rinderpest and the cessation of RPV vaccination, the presence of PPRV in cattle may indicate that the absence of cross-protective immunity has facilitated the spread of PPRV into atypical hosts. Hence, an enhanced immunosurveillance of both domestic and non-domestic species may establish the potential reservoir species that should be targeted during future PPRV eradication campaigns. Accordingly, we screened cattle sera from three villages in Northern Tanzania for the presence of neutralising antibodies against PPRV, CDV and RPV (Fig. 5). Of 125 serum samples screened, 4 samples were identified with PPRV-specific neutralising activity (samples 83, 155, 211 and 236). Surprisingly, we also detected 5 samples with RPV-specific activity (56, 70, 118, 170 and 192) and a further two samples (125 and 197) with high activity against RPV and cross-neutralising activity against PPRV. Further, 2 samples neutralized CDV specifically (samples 195 and 204). Hence, we were able to confirm the exposure of cattle to PPRV (4/125, 3.2%), an RPV-like virus (7/125, 5.6%) and CDV (2/125, 1.6%). Given the cessation of rinderpest vaccination in Tanzania in 1998 and the confirmation of rinderpest eradication in Tanzania in 2003, the detection of RPV-specific antibodies in 2009 is of significant interest. The samples that displayed RPV-specific neutralisation were screened against an extended panel of morbillivirus pseudotypes including MeV and PDV, and compared with sera raised against MeV, CDV, PPRV or RPV (Fig. 6). Human serum from an MMR-vaccinate neutralized MeV efficiently and displayed good cross-neutralisation of RPV and PPRV, with weak activity against CDV (Fig. 6a). Conversely, serum from an RPV-vaccinated animal neutralized RPV most efficiently but displayed cross-neutralisation of CDV, PPRV and MeV (Fig. 6a). The sera from a CDV-vaccinate and a PPRV-vaccinate neutralized their respective homologous viruses most efficiently with weaker cross-neutralisation of other morbilliviruses at high serum concentrations (Fig. 6a). In contrast, the Tanzanian cattle sera (170, 192, 56 and 188) neutralized RPV primarily with only weak cross-neutralisation of MeV, PPRV or CDV. As the neutralising activity in these samples was modest in comparison with the sera from cattle exposed to RPV experimentally (Figs. 1 and 4), the specific neutralisation of RPV by these samples may reflect lower antibody titres in the sera *per se*. However, the specificity of the sera for RPV may also indicate that the virus to which they were exposed is a genetic outlier, most closely related to RPV but substantially divergent from MeV, CDV and PPRV. Given that all the sera we have tested to date displayed a higher titre of neutralising antibody against their homologous morbillivirus, the data suggest that these Tanzanian cattle were exposed to an RPV-like bovine morbillivirus.

## 4 Discussion

The OIE has identified PPR as a global priority for eradication. PPRV continues to spread globally [35], [36], with over 1 billion sheep and goats at risk from infection, approximately 80% of the world’s small ruminants [37]. RPV eradication led to the cessation of vaccination and, as a result, there are fears that morbilliviruses such as PPRV or CDV could spill over into the global population of 1.5 billion immunologically-naïve cattle [38]. Indeed, PPRV seropositive cattle have now been identified in countries across Africa and Asia [39], [40], [41], [42]. Understanding the patterns of morbillivirus infections in domestic livestock and wildlife populations will be critical to ensuring the success of future global eradication programs.

To validate the sensitivity of the pseudotype-based assay for PPRV neutralising antibodies, we screened sera from three groups of cattle, one infected with a wild type strain of PPRV (IC89) and two vaccinated with live attenuated PPRV vaccines (N75 and S96). Animals infected with IC89 developed significantly higher antibody titres, antibodies that cross-neutralized PPRV strains from each of the four lineages. These data suggest that an animal exposed to a virus such as IC89, and which had recovered and generated a strong humoral response, would be cross-protected from infection with viruses from all four lineages. In contrast, the neutralising responses of the sera from animals vaccinated with N75 or S96 generated significantly lower titres of neutralising antibodies in comparison with the IC89-infected animals. Lineage 2 N75 and lineage 4 S96-containing vaccines are licenced in several PPRV-endemic countries. Neutralising antibody titres induced by the two vaccines were highest against the vaccine strain N75, and lowest against lineage 3 Kenya 2011. Such observations may assist the design of future eradication strategies and retrospective analyses of sera from vaccine trials might provide an insight into whether the level of neutralising antibody induced by vaccination correlates with immunity to infection with viruses from distinct lineages. In this study, we were restricted to the examination of sera from cattle infected experimentally with PPRV. Future studies should examine sequential sera from goats and sheep, the primary targets for PPRV vaccination campaigns, as it is likely that the replication of live attenuated viruses will vary between target species and hence the strength and breadth of immunity induced will vary accordingly.

The high sensitivity of the pseudotype-based assay facilitated the detection of a higher proportion of sero-positives than a widely used ELISA. While it should be noted that the ELISA was optimised for specificity, allowing PPRV exposure in a herd to be confirmed, it was evident that the assay sensitivity was low, underestimating the number of positive animals significantly. Indeed, three animals with high titre neutralising antibodies tested negative by ELISA. Presumably the humoral response in those animals targeted epitopes on PPRV H distinct to that recognised by the monoclonal antibody within the kit or targeted the PPRV F protein.

We detected PPRV neutralising responses in sera from both buffalo and goats. The presence of neutralising antibodies in 38% of buffalo sera that had tested negative previously by ELISA suggests that PPRV exposure was under-reported in previous studies [34]. The presence of PPRV in Serengeti buffalo is consistent with findings of PPRV antibody-positive wildlife species in the vicinity [43]. The detection of CDV-specific antibodies in 5 of the buffalo indicates that morbillivirus exposure in wildlife is not restricted to PPRV and is consistent with findings of CDV seropositivity in lions within the same ecosystem [44]. The co-circulation of PPRV and CDV in the same livestock and wildlife species may interfere with the accurate diagnosis of PPRV infection using existing serological techniques. The pseudotype-based neutralisation test may offer additional, rapid discrimination of findings.

By comparing batches of experimental sera from animals exposed to either RPV or PPRV, we demonstrated that the neutralising responses were highest against the homologous virus, consistent with the original serology-based evidence for the distinction of RPV and PPRV [45], [46], [47], [48]. Accordingly, we are able to examine banks of sera for historical exposures to morbilliviruses and to predict the nature of the infecting agent. Using this approach, we demonstrated that cattle in three villages in Tanzania had been exposed to not only PPRV, but also CDV, consistent with the earlier findings with buffalo sera. Intriguingly, seven animals had neutralising antibodies targeted primarily against RPV, two of which also cross-neutralized PPRV efficiently. Given that (a) rinderpest vaccination ceased in this region in 1997, (b) three of the seven animals were <5 years of age and (c) positive animals were detected in cattle from three separate villages, the presence of anti-RPV antibodies in 2009 suggests a recent exposure to an RPV-related bovine morbillivirus. There is a compelling precedent for the existence of an RPV-related bovine morbillivirus in cattle populations. In 1975, a “bovine paramyxovirus” was isolated from cattle displaying symptoms of sporadic encephalomyelitis in Germany and Switzerland [49]. Serological tests on cells infected with the agent confirmed reactivity with sera raised against RPV, or convalescent sera from measles patients with sub-acute sclerosing panencephalitis (SSPE) [49]. In 1976, a morbillivirus was identified in cattle affected with malignant catarrhal fever in Colorado [50]. Immunofluorescence studies of tissues from an infected animal reacted strongly with serum from an animal infected with the Kabete “O” strain of RPV but only weakly with sera raised against MeV or CDV [50]. In 1998, an investigation of sporadic outbreaks of non-suppurative meningoencephalomyelitis in Swiss cattle noted that four cases displayed immunoreactivity with monoclonal antibodies raised against either RPV or CDV nucleocapsids [51]. The existence of an RPV-related bovine morbillivirus may also explain why an animal in a previous rinderpest vaccine trial displayed neutralising antibodies against RPV prior to vaccination [52]. Given that recent studies have revealed the astonishing diversity of paramyxoviruses circulating in bats and rodents [53], we should expect that many more paramyxoviruses await discovery in other species. Indeed, a morbillivirus of domestic cats has recently been described, a virus that appears to be relatively widespread globally [54], [55], [56]. The data presented here may suggest an additional bovine morbillivirus is circulating, with possible implications for PPRV diagnostics, surveillance and vaccination.

If vaccination to eradicate PPR is to succeed, future studies should address which species act as reservoirs of infection and whether they may shed infectious virus. If species such as cattle shed infectious PPRV, this must be taken into consideration in a post-PPRV eradication environment. Continued immunosurveillance will ascertain whether PPRV is spreading more widely; broadening its host species in a post-RPV world. Finally, we should consider whether RPV eradication has created a vacated niche [57] for PPRV other morbilliviruses such as CDV, or a novel RPV-related bovine morbillivirus.
