# Supplementary material for: Enhanced immunosurveillance for animal morbilliviruses using vesicular stomatitis virus (VSV) pseudotypes
Source: Vaccine. 2016 Nov 11;34(47):5736–43. doi: 10.1016/j.vaccine.2016.10.010 (PMC5084683; doi:10.1016/j.vaccine.2016.10.010)
Supplement: Supplementary data 1 [file mmc1.docx]

**Supplementary methods**

**PCR Amplification of the H and F genes of animal morbilliviruses.** PCR amplifications used the following primers:

**PPRV:** haemagglutinin (H) gene first round PCR using primers Kenya20115primeUTRH    5’-GACGAAAGGTTAGTCACCATG-3’ and Kenya20113primeUTRH     5’-GCAGCTCTGCTCAGA-3’ followed by second round PCR using primers PPRVmultipleHnotFwd     5’-GTAGCGGCCGCATGTCCGCACAAAG-3’ and Nig75bamrev                         5’-GGGGGATTCTCCTCAGACTGGATTACATGTT-3’. Fusion (F) gene first round PCR utilized primers Kenya2011PPRVF5UTR       5’-CACATTCATGCACAAACATCATG-3’ and Kenya2011PPRVF3UTR       5’-GGATGATTCGGGCTATTCTGATTA-3’ followed by second round PCR using primers PPRVmultipleFnotFwdshort   5’- GTAGCGGCCGCATGACACGGGTCGCAA – 3’ and

PPRVmultipleFbamRev             5’ – GTAGGATCCCTACAGTGATCTCACG – 3’.

**Measles virus (MeV):** Measles virus H and F were amplified from RNA prepared from the Edmonston vaccine strain using the primers Sal1FWD   5’-GTTAGTCGACATGTCACCGCAACGAGACC-3’ and Not1REV   5’-GACTGCGGCCGCCTATCTGCGGTTGGTTCCATCTTC-3’.

**Phocine distemper virus (PDV):** H and F were amplified from NLD88 RNA [[31](#_ENREF_31)] using primers PDV H SAL F WT: 5’-CCCGTCGACACCATGTTCTCTCATCAAGACAAG-3’ and PDV H NOT REV: 5’-GGGGCGGCCGCTTAAGGATCAAGACGATCAC-3’, and PDV F sal1 Fwd 5’-GGGGTCGACACCATGACTCGAGTCAAGAAAC-3’ and PDV F not1 Rev delta 633 5’-Gggcggccgcttgttatcgctttttacagcaac-3’.

CDV H and F were amplified as described previously [[30](#_ENREF_30)].

All amplifications were performed with the following thermocycling conditions: denaturation at 94^o^C for 5 minutes, followed by 35 cycles of 94^o^C for 30 seconds, annealing at 50^o^C to 64^o^C (depending on primer Tm) for 60 seconds and extension at 72^o^C for 120 seconds, with a final extension at 72 ^o^C for 10 minutes.
